# Veterinary World reviewer acknowledgment 2017

**DOI:** 10.14202/vetworld.2018.10-13

**Published:** 2018-01-12

**Authors:** A. V. Sherasiya and Nazir

**Affiliations:** Veterinary World, Star, Gulshan Park, NH-8A, Chandrapur Road, Wankaner – 363621, Dist. Morbi, Gujarat, India

## Contributing reviewers

Veterinary World editorial team would sincerely like to thank all of our reviewers who contributed to peer review for the journal in 2017.

A. Aygun Turkey

A. K. M. Anisur Rahman Bangladesh

Abdelaziz ED-DRA Morocco

Abid Hussain Shahzad Pakistan

Ahmed Ali Mahmoud Saleh Japan

Ahmed S Abdoon Egypt

Ailian Geng China

Alfredo Medrano Mexico

Amira Taman Egypt

Amlan K. Patra India

Ammar Ahmad Khan Pakistan

Ana Paula Loureiro Brazil

Anand S. P. India

Andres Carvajal Colombo

Anna Faltýnková Czech Republic

Annamaria Passantino Italy

Antoni Prenafeta Spain

Antonio Varcasia Italy

Anut Chantiratikul Thailand

Arathy D. S. Nair USA

Arbouche Rafik Algeria

Archana Jain India

Archana Verma India

Arda Sozcu Turkey

Arda Yildirim Turkey

Aripudur Sellappan Rajendiran India

Arun Kumar De India

Arya Sobhakumari USA

Ashish Chopra India

Ashwani Kumar India

Aspinas Chapwanya Kenya

B. Madhusudhana Rao India

Bala Manickam USA

Balamurugan Vinayagamurthy India

Bambang Pontjo Priosoeryanto Indonesia

Bartosz Kierończyk Poland

Ben Adler Australia

Bersissa Kumsa Eseta Ethiopia

Brad M Isaacson USA

Bryony A. Jones UK

Buket Er Demirhan Turkey

C. E. Atkins USA

Chaleow Salakij Thailand

Chanchal Guha India

Chandramani Pathak India

Christian Tiambo Keambou Kenya

Christopher Didigwu Nwani Nigeria

Chyer Kim USA

Claudia Bezerra da Silva Brazil

Coen M. Adema USA

Cromwell Purchase Qatar

David Kennedy USA

Deepti Chachra India

Denisa Soledad Pérez Gaudio Argentina

Dinesh M. D. India

Dragica Salamon Croatia

Duane L. Garner Garner USA

E. Carretón Spain

Ehsan Ahmadpour Iran

Elif Çelik Turkey

Elisabetta Manuali Italy

Emilia Bagnicka Poland

Erma Safitri Indonesia

Eryk Abdul Rantam Indonesia

Fabio De Rensis Italy

Felipp Silveira Ferreira Brazil

Filippo Giarratana Italy

Florence D Tarfa Nigeria

Fufa Ido Gimba Malaysia

Gamal Wareth Germany

Genevieve Andre-Fontaine France

Gitao Chege George Kenya

Giuseppe Piccone Italy

Gnanavel Venkatesan India

Guilherme Dias de Melo France

Guillermo Roberto Risatti USA

Gul Ahmad USA

Guoxiang Chao China

Guo-zhong Zhang China

Gwendolyn L Carroll USA

Hanna Markiewicz Poland

Hari Mohan Saxena India

Hasan Meydan Turkey

Hassan Borji Iran

Hazem Shaheen Egypt

Hua-Ji Qiu China

Hudaa Neetoo Mauritius

Hudson A. Pinto Brazil

Hugo Leonardo da Cunha Amaral Brazil

Huiling Sun China

Ionel D. Bondoc Romania

Irena Musik Poland

Ismail Hakkı Tekiner Turkey

James Satvil Dalis Nigeria

Jason Devoy Keegan Ireland

Jayraw A. K. India

Jebreil shams shamseddin Iran

Jerome Andonissamy India

Jitendra Goswami India

João Simões Portugal

Jose Edmundo Apraez Guerrero Colombia

Joseph Bosilevac USA

Joshua Oluoch Amimo Kenya

K. G. Meade Meade Ireland

Kai Huang USA

Kannan Thandavan Arthanari India

Karim El-Sabrout Egypt

Kirstine Klitgaard Schou Denmark

Kolluri Gautham India

Kosta Y. Mumcuoglu Israel

Koushlesh Ranjan India

Laurent Falquet Switzerland

Laxmi Narayan Sarangi India

Lenin Lenin Aguirre Riofrio Ecuador

Liben Chen USA

Liliana Aguilar-Marcelino Mexico

Linous Munsimbwe Zambia

Livia Ribeiro Mendonca Brazil

Luis Souza Lima de Souza Reis Brazil

Mahmoud Mohey Elhaig Egypt

Manjunatha Bodhaganahalli Oman

Manjunatha Narayanappa Belaganahalli USA

Maria Emilia Eirin Argentina

Mário Manuel Dinis Ginja Portugal

Martin M Nyaga South Africa

Masayuki Shimada Japan

Md Fakruddin Bangladesh

Mehmet Fatih Aydin Turkey

Mian Muhammad Awais Pakistan

Michael M. Schutz USA

Miguel Quaresma Portugal

Minakshi Prasad India

Mobini Behzad Iran

Mohamed Haroun - Haroun Qatar

Mohammad Mijanur Rahman Malaysia

Mohammad Taghi Rahimi Iran

Mohammed Hamdy Farouk China

Mohan Maddur USA

Mohie Abdallah Mahmoud Haridy Japan

Moustafa kardjadj Algeria

Moutaz Zarkawi Syria

N. De Briyne Belgium

N. Punniamurthy, Natesan India

Nadeem Shabir India

Naim Deniz Ayaz Turkey

Nazarudin M.F. Malaysia

Nazli Ercan Turkey

Nicholas Johnson UK

Nirupama Agrawal India

Nitesh Kumar India

Noha Salem Egypt

Nuh Kiliç Turkey

Oludayo O Fashina South Africa

Osama Ibrahim Azawi Iraq

Panagiotis E Simitzis Greece

Panch Kishor Bharti India

Parag Nigam India

Paramanandham Krishnamoorthy India

Pinaki Prasad Sengupta India

R. Mirmahmoudi Iran

Rachel BINET USA

Rafael Cardoso Carvalho Brazil

Rafiqul Islam India

Rahul Nandre USA

Rajani Ravindranath Thanissery USA

Raquel Salazar Lugo Venezuela

Ravi Kant Upadhyay India

Rekha Sharma India

Reza Masoumi Iran

Richa Sood India

Rodrigo Alberto Jerez Ebensperger Spain

Rolf karl Schuster United Arab Emirates

Rui Wu China

S. R. Rüegg Czech Republic

Sameer D Pant Australia

Sandeep Ghatak India

Satoshi Sekiguchi Japan

Seyyed Khalil Hosseinihashemi Iran

Shailbala Singh USA

Shoja Jafari Iran

Silvana Scarcella Argentina

Sinan Ince Turkey

Sorin Daniel Dan Romania

Sreekumar Chirukandoth India

Sreenu Makkena India

Stefania Perrucci Italy

Süleyman Cilek Turkey

Sunil More USA

Sven Kaese Germany

T. Madani Toufik Algeria

Tanko Polycarp Nwunuji Nigeria

Thomas J Nolan USA

Tomas Erban Czech Republic

Ummey Shameem India

V. C. Rayulu India

Valeria De Cesaris Italy

Varij Nayan India

Vera Mores Rall Brazil

Vihang Vithalrao Patil India

Vishal S Suthar India

Walied Abdo Egypt

Widya Paramita Lokapirnasari Indonesia

William Alberto Cañón-Franco Colombia

Wojciech Barański Poland

Ya-Ching Lin Taiwan

Ya-Ling Huang Taiwan

Yasser Mahmmod Denmark

Youssef A. Attia Saudi Arabia

Yukita Sato Japan

Yves Millemann France

Zahid Naseer Pakistan

Zeki Aras Turkey

Zuhair Ismail Jordan

Zygmunt Kazimierz Giżejewski Poland

